# Culture shapes how we describe facial expressions

**DOI:** 10.1038/s41598-024-72432-w

**Published:** 2024-09-16

**Authors:** Ewelina Wnuk, Jan Wodowski

**Affiliations:** Faculty of Modern Languages, University of Warsaw, 00-312 Warsaw, Poland

**Keywords:** Psychology, Anthropology

## Abstract

From Darwin through Wittgenstein to contemporary scientific investigations, it has been argued humans tend to view facial expressions through a mentalistic lens. According to this view, when looking at someone’s expressive face, we see emotion and are unable to describe the face in behavioral terms, i.e., name the details of facial movements. At the same time, however, a growing body of work shows cultures in fact differ in the degree of importance they attribute to mental states and willingness to discuss them. Is this variation reflected in the linguistic coding of facial expressions? To explore this, we conducted two facial expression naming tasks targeting mental states and facial movements with speakers of Maniq (Austroasiatic, Thailand) and Polish (Indo-European, Poland), representing highly diverse linguistic and cultural settings. We found that, while Polish speakers conformed with the predicted orientation towards mental states, this did not hold for Maniq speakers. The Maniq were instead oriented towards behavioral aspects of faces, naming them more frequently, more precisely, and with higher consensus, compared to the Polish. These differences are carved into the Maniq and Polish lexicons, suggesting diverse cultures exhibit differential specialization in verbalizing expressive faces.

## Introduction

For generations, prominent scholars have argued facial expressions are by default associated with internal mental states and are easiest to describe in such terms. Describing physical details of facial expressions, on the other hand, is considered difficult. As noted by Darwin^1^: “No one, I believe, can clearly describe a sullen or sly expression; yet many observers are unanimous that these expressions can be recognized in the various races of man”. Almost a century later, Wittgenstein made a similar point, suggesting expressive faces are viewed and verbalized primarily through the lens of emotion, not facial movements: “We see emotion (…) We do not see facial contortions and make inferences from them (like a doctor framing a diagnosis) to joy, grief, boredom. We describe a face immediately as sad, radiant, bored, even when we are unable to give any other description of the features”^2^.

Similar views regarding ineffability of the physical aspects of faces are found today. Faces are considered a prime example of a domain which eludes precise description (cf. ref.^3^). This point has been made for both facial features as well as facial movements. In the context of eyewitness accounts, for instance, it is commonly acknowledged obtaining detailed person descriptions is difficult because of the “limited vocabulary that individuals have for describing the human face”^4^. Facial expressions of emotion, similarly, are considered challenging to describe with precision “Although observers can articulate individual features (…) such as the down turned mouth in an unhappy face, these descriptions are often incomplete and fail to capture the subtleties”^5^.

Thus, fine details of facial expressions, such as specific facial movements, seem to resist description and elaborate vocabularies seem to be lacking. Talking about emotion and other mental states, on the other hand, appears more straightforward, especially from the perspective of the well-described large languages of industrialized societies, which have dozens or even hundreds of emotion terms^6,7^ and whose speakers have no special difficulty in classifying facial expressions with mental state terms^8–10^. This would suggest expressive faces might be easier to talk about in mentalistic rather than behavioral^11^ terms, in line with the evolved social cognitive function of facial expressions^1,12, 13^.

At the same time, however, a growing body of cross-cultural work suggests the extent to which people actually talk about mental states is culturally variable. There are multiple reports of communities, e.g., Ceq Wong speakers of Malaysia, Dagbani and Fante speakers of Ghana, with relatively small mental state vocabularies, in which mental state talk and psychologizing are uncommon^14,15^. Similarly, extensive work in linguistics shows emotion descriptions in many languages are dominated by terms which do not denote mental states, but are descriptions of bodily sensations and actions, both conventionalized figurative terms and literal expressions^16–21^. Finally, also an increasing number of experimental studies shows some cultures, e.g., the Hadza of Tanzania^22,23^, the Himba of Namibia^24^, and the Trobrianders of Papua New Guinea^25^, frequently describe expressive faces not just with emotion and other mental states terms, but also terms for actions^9^. These findings point to a more general fact emotion concepts are culturally variable and can be differentially constituted^26,27^. So, while mental states are a central aspect of emotion in the Euro-American context, in other cultures other aspects of emotion, e.g., bodily experience and action, are foregrounded^15,22^.

In addition, some societies specifically avoid discussing mental states of other people^28,29^. In her classic ethnography *Coming of Age in Samoa*, Margaret Mead describes how Samoans persistently refuse to speculate about mental lives of other people^30^. Underlying this attitude is a conviction that it is impossible to know what others are feeling or thinking. In the words of Samoans: “we cannot know what is in another person’s depths”^31^. Similar beliefs have been widely described throughout the Pacific^29,32,33^, including by interdisciplinary teams of ethnographers and experimenters^34^, suggesting considerable cross-cultural diversity in the willingness to talk about other people’s mental states^28,35, 36^ and in the onset of mental state reasoning in childhood^34^.

The above variation puts into question the suggestion that mentalistic descriptions are a default way of verbalizing facial expressions, or that they are universally easy. If talking about mental states is culturally constrained, or if mental states as such are not culturally salient, it would not seem surprising if languages differed in how well they coded different aspects of expressive faces. Yet, while this seems plausible, this question has not been examined directly.

In this article, we investigate it for the first time by comparing the naming of facial movements and emotions on the face. More specifically, we consider the expressibility (or codability) of facial movements and emotions in language. As observed by the linguist Charles Hockett, “languages differ not so much as to what can be said in them, but rather what is relatively easy to say” (in ref.^37^). Codability speaks to this observation, capturing ease of description on a continuum. For example, a recent investigation into the language of perception has found languages differ substantially as to which sensory domains are most richly linguistically coded and which ones are most ineffable^38^. This finding challenges the assumption that the codability of human perceptual impressions follows a single Aristotelian-like hierarchy, with vision at the top followed by audition, smell, touch, and taste, suggesting instead that the ordering of the senses is culture-specific.

We ask a similar question for faces: Does codability of expressive faces differ cross-linguistically? In addition, does it vary depending on what aspects of faces are in focus (facial movements vs. emotion)? Given that mentalistic descriptors are scant or infrequent in some languages, it is possible these languages could instead be tailored to talking about facial expressions in behavioral terms, i.e., naming facial movements. If, on the other hand, details of facial movements are indeed ineffable^5^, i.e., subject to a general expressibility constraint, they might be difficult to name in all languages, including those with small emotion vocabularies.

**Fig. 1:**
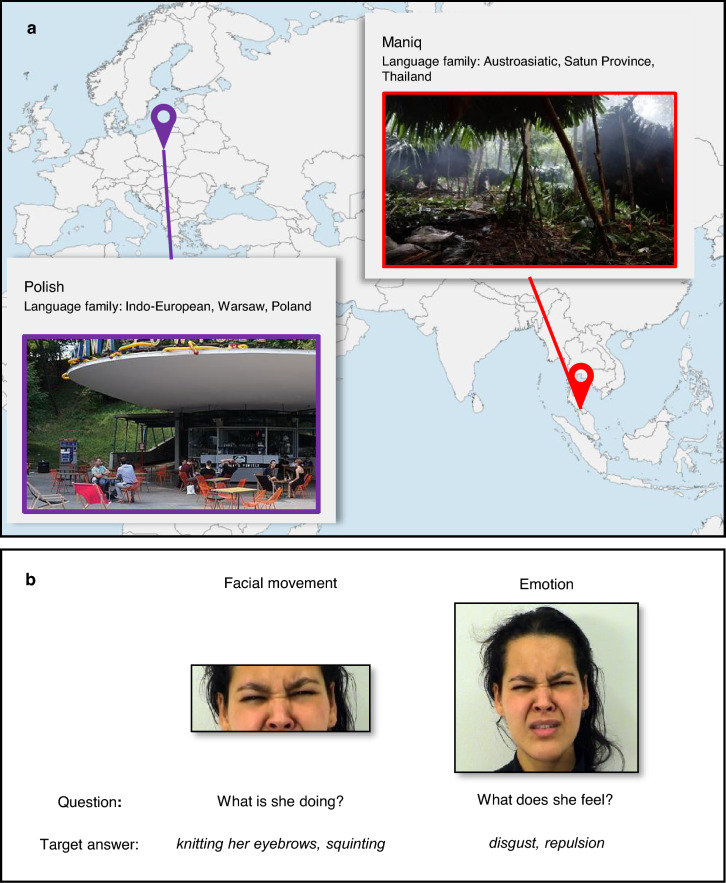
Data collection sites and experimental tasks (**a**) Data collection sites. The study groups were urban-dwelling Polish speakers in Warsaw and the forest-dwelling Maniq speakers in the Banthad mountains, Satun Province, Southern Thailand. Map created with www.mapchart.net under CC BY-SA 4.0 Deed. Images: (1) Left—By Cyrkiel-network, CC0, via Wikimedia Commons and (2) Right—By Ewelina Wnuk (**b**) Still frames from a video clip illustrating the two types of task stimuli, experimental questions, and target answers (for the full list of stimuli, see Supplementary Tables S1 and S2). Adapted from the Amsterdam Dynamic Facial Expression Set^60^ (reproduced with the Authors’ permission).

To explore these questions, we investigated the linguistic coding of expressive faces in two naming tasks: one focused on individual facial movements (facial movement task) and one on facial configurations (emotion task), designed to elicit, respectively, behavioral and mentalistic descriptions. We targeted Maniq, an Austroasiatic language spoken by a forest-dwelling hunter–gatherer group of Southern Thailand (Fig. 1A), known from prior work for its rich verbal lexicon with a high level of detail in certain types of action descriptions^39,40^, including some facial actions^41^. Like its close relative—Ceq Wong^14^—Maniq has a relatively small emotion lexicon. We compared Maniq to a representative of an Indo-European family—Polish (for extended descriptions of study populations, see Supplementary Information), which for aspects relevant here is largely similar to English and many other large languages of industrialized societies, i.e., it has a large emotion lexicon of a few hundred terms, readily applied to facial expressions^7,10, 42,43^. The two study groups, therefore, represented highly diverse cultural and linguistic settings.

We targeted codability because it is a good indicator of what aspects of faces are easily accessible to consciousness and amenable to linguistic expression^38^. Beyond merely indexing linguistic diversity, differences in codability have wider implications for communication and cognition. For instance, codability may reflect differential culture-specific emphasis on different domains, showing that even fields previously considered inaccessible to language like smell can be linguistically elaborated in some cultural contexts^44,45^. Different facets of codability, e.g., availability of dedicated linguistic labels, are related to communicative efficiency^46,47^ and can have non-trivial consequences for perception^48,49^, memory^50^, and other aspects of thought^51^. Thus, potential differences in the codability of expressive faces would generate implications for communication and cognition^52–55^, and add to the body of work documenting cross-cultural diversity in the processing of expressive faces^56–58^.

To test codability, we presented Maniq and Polish speakers with two sets of brief video stimuli: a selection of action units (individual components of facial expressions, as specified in FACS—the Facial Action Coding System^59^) and dynamic emotional expressions^60^ (for the full list of stimuli, see Supplementary Tables S1 and S2), to test the naming of facial movements and emotions, respectively. As illustrated by the examples in Fig. 1B, action units were movements of individual muscles or groups of muscles in one face area, e.g., knitting eyebrows, squinting, etc., whereas emotional expressions involved configurations of facial movements on the entire face, e.g., a disgusted face, an angry face, etc. Instructions, provided in participants’ native language, targeted mental states for the emotion task (What does she/he feel?) and actions for the facial movements task (What is she/he doing?). While our study does not center on perceptual models of face processing, it is relevant to mention that facial expressions are processed by a partially separate perceptual system, interacting with the system for identities^61,62^. Some^63^, though not all^64^, facial movements can support identity recognition under certain circumstances. Most pertinent to our study, however, faces are associated with holistic processing (i.e., they are processed as unified wholes), but various experimental manipulations (e.g., presenting two misaligned face halves) can reduce or inhibit holistic processing^65^. Thus, in addition to the two tasks differing by instructions, the presentation of whole faces and their subparts may have differed at a perceptual level. Data collection and coding were carried out by researchers fluent in test languages and familiarity with the cultural setting. The test procedure was the same for Maniq and Polish speakers, with the order of tasks counterbalanced across participants. Participants were free to provide as many responses as they wished. Our goal was to elicit spontaneous language applied to facial expressions from each group. Specifically, we wanted to know whether the two languages would differ in their coding of different aspects of expressive faces.

To evaluate that, we first coded the data from both tasks by identifying the main content responses, i.e., elements carrying the semantic gist of each response. We omitted function words, grammatical morphemes, and modifiers, and extracted the common roots of morphologically related responses (for detailed response coding procedure, see Supplementary Information). For instance, a description provided by a Maniq participant *baʔɛt, ʔɛʔ pimikluk ɡanaʔ* ‘good/happy, he’s smiling at his partner’, was coded as two main responses: *baʔɛt* ‘to be good/happy’ and *pikluk* ‘to smile’. We then classified each response according to type of description, i.e., “bodily action” (e.g., open one’s mouth, look, move one’s head forward) or “mental state” (e.g., afraid, anger, think). This classification draws on the graded continuum of social inference^29^ employed in previous work^23^, with the coded categories of bodily actions and mental states reflecting the two endpoints of the continuum. Intermediate cases, whereby both mental and bodily aspects were involved, were classified consistent with their primary denotative meaning, e.g., *kahey* (Maniq)*/płakać* (Polish) ‘to cry’ and *pikluk (Maniq)/śmiać się* (Polish) ‘to laugh’ were coded as “bodily action”. A small number of responses which did not fit clearly into either category, e.g., *campɨs* ‘to be ill’ (Maniq), *potwierdzać* ‘to confirm’ (Polish), *hay wac* ‘like a squirrel’ (Maniq), *nic* ‘nothing’ (Polish), were coded as “other”. Exceptions to the general coding rules and difficult cases are described in the response coding procedure in Supplementary Information.

## Results

### Types of responses

**Fig. 2:**
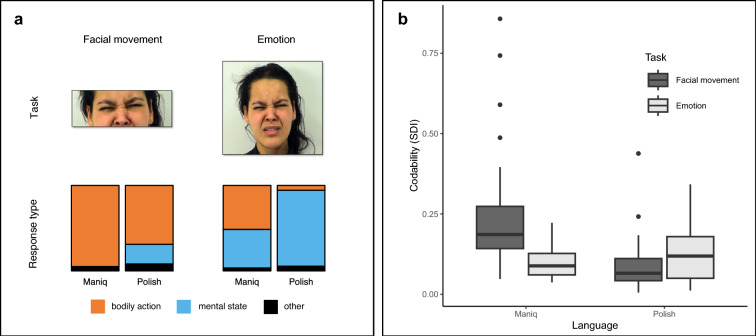
Results: Types of responses and codability. (**a**) Types of responses given by the Maniq and Polish speakers in the facial movement and emotion task. The mosaic plots show the overall proportion of bodily action, mental state, and other responses, across each task. Across tasks, Maniq speakers provided more bodily action responses than Polish speakers, while Polish speakers provided more mental state responses than Maniq speakers. Stimuli adapted from the Amsterdam Dynamic Facial Expression Set^60^ (reproduced with permission) (**b**) Boxplots of codability scores, measured with Simpson’s Diversity Index (SDI), across the two experimental tasks for Maniq and Polish. In Maniq, codability was higher for facial movements than for emotions, whereas in Polish there was no difference. Codability of facial movements was also higher for Maniq than for Polish. The center lines within the boxes mark the medians, the boxes extend from the first to third quartiles, the whiskers and individual data points mark the range of obtained values.

To see how different aspects of expressive faces are expressed in each language, we first considered what types of responses (“bodily action” vs. “mental state” vs. “other”) were provided in each task. Figure 2A provides a summary of the overall proportions of different types of responses for each group. As expected—given that the tasks targeted different types of percepts—both Maniq (χ^2^ (2, *N* = 1325) = 421.590, *p* < 0.001, *V* = 0.564, 95% *CI* [0.511, 0.619]) and Polish speakers (χ^2^ (2, *N* = 1164) = 484.120, *p* < 0.001, *V* = 0.645, 95% *CI* 0.588, 0.703]) gave different types of responses across the two tasks (for the full list, see Supplementary Table S3). Despite this general similarity, however, there were clear differences across groups in the types of responses given in the facial movement task (χ^2^ (2, *N* = 1533) = 208.360, *p* < 0.001, *V* = 0.369, 95% *CI* 0.320, 0.419]) and the emotion task (χ^2^ (2, *N* = 956) = 229.500, *p* < 0.001, *V* = 0.490, 95% *CI* [0.428, 0.554]). Maniq speakers revealed a tendency to describe stimuli in behavioral terms, i.e., name bodily actions. This was true not only for the facial movement task (Maniq 95% vs. Polish 69%), where such responses were targeted by the instruction, but also for the emotion task (Maniq 51% vs. 6% Polish). Polish speakers, on the other hand, showed a greater tendency to respond in mentalistic terms, i.e., name mental states. This was true not only for the emotion task (Polish 89% vs. Maniq 45%), where such responses were targeted by the instruction, but also the facial movement task (Polish 23% vs. Maniq 1%). Thus, even though the instructions placed emphasis either on mental states or actions, Maniq and Polish participants revealed distinct patterns in their responses, with Maniq speakers focusing more on bodily actions and Polish speakers on mental states.

### Codability of expressive faces

We next considered the codability of expressive faces, i.e., how easy it is to express facial stimuli in language. We followed the operational definition in Majid et al.^38^, where codability is defined as “the degree to which a stimulus was consistently named within a language community”. This operationalization draws on Brown and Lenneberg^66^, who originally proposed the notion of codability and showed that, amongst a variety of correlated measures reflecting codability, cross-speaker naming consistency captures this notion best. We used an established measure of cross-speaker naming consistency—Simpson’s Diversity Index (SDI)^38,45,67–69^—which takes into account type and frequency of responses provided for a given stimulus. SDI is computed using the formula: $SDI=\sum n(n-1)/N(N-1)$, where n is the total number of times a particular response was given for a stimulus, and N the total number of all responses for that stimulus. An SDI of 1 indicates complete agreement, i.e., all participants gave the same response, and an SDI of 0 indicates complete disagreement, i.e., all participants gave different responses (for details on determining same and different responses, see Response coding procedure in Supplementary Information).

Raw SDI scores were normalized through log-transformation and were subsequently analyzed with linear mixed-effects modeling in R^70,71^. The full model included fixed intercepts for language, task, and the interaction between them, as well as a random intercept for stimulus (for summaries of full models, see Supplementary Tables S4 and S5). Random effect estimation indicated that 29% of the total residual variance in normalized SDI scores was due to stimulus-level variability. To test for the main effects of independent variables, likelihood ratio tests were used comparing models with and without each fixed intercept. We found a significant difference in agreement between Maniq and Polish (χ^2^ (1) = 16.22, *p* < 0.001) and an interaction between language and task (χ^2^ (1) = 17.93, *p* < 0.001), but no difference between tasks (χ^2^ (1) = 2.87, *p* = 0.090) (see Fig. 2B), meaning that not only the overall agreement levels were higher in Maniq than in Polish, but also that each language exhibited a different pattern of specialization across the two tasks. Specifically, facial movements were more codable in Maniq than in Polish (*B* = 0.907, *SE* = 0.118, 95% *CI* [0.675, 1.138], *t*(60) = 7.678, *p* < 0.001), while emotional expressions were equally codable in both languages (*B* = − 0.026, *SE* = 0.171, 95% *CI* [− 0.361, 0.310], *t*(60) = − 0.152, *p* = 0.880). Facial movements were also more codable than emotional expressions in Maniq (*B* = 0.702, *SE* = 0.174, 95% *CI* [0.361, 1.044], *t*(110.775) = 4.029, *p* < 0.001), but they were equally codable in Polish (*B* = − 0.230, *SE* = 0.174, 95% *CI* [− 0.572, 0.111], *t*(110.775) = − 1.322, *p* = 0.189) (for summary statistics of codability scores, see Supplementary Table S6).

The results suggest that, even though details of facial expressions are considered difficult to put into words, this difficulty is not universally present in all languages. Maniq speakers express facial movements with greater ease and—contrary to the assumption that faces tend to be described in mentalistic terms—they exhibit a preference for behavioral facial descriptions. Conversely, Polish speakers have a preference for mentalistic descriptions, although they do not show higher codability in naming facial configurations.

This is a novel finding as prior work targeting facial expressions of emotion typically considered basic emotion perception, as predicted by the universality hypothesis^72^, and did not evaluate labeling agreement directly. Considering emotion recognition with emotion terms for our dataset, the twelve stimuli depicting the six basic emotions (joy, sadness, anger, fear, surprise, disgust) were consistently labeled with the expected emotion words by higher percentages of Polish speakers (*M* = 0.776, *SEM* = 0.051, 95% *CI* [0.663, 0.889]) than Maniq speakers (*M* = 0.269, *SEM* = 0.102, 95% *CI* [0.045, 0.493]) (two-tailed Mann–Whitney test, *U *= 20.5, *n*_*1*_ = *n*_*2*_ = 12, *p* = 0.003, *r* = 0.72, 95% *CI* [0.40, 0.88]) (see Supplementary Table S7 for detailed results for each stimulus). However, as shown here this did not lead to higher codability, since Polish speakers employed a high number of synonymous or semantically related responses impacting codability negatively. Accurate emotion recognition thus does not automatically translate to high codability.

To sum up, Maniq and Polish data presented here demonstrate that languages do differ in their coding of expressive faces, with Maniq exhibiting a higher codability of facial movements. To shed further light on the nature of this difference, we now zoom in on another facet of codability—availability of dedicated lexical resources.

### Dedicated lexicon for facial movements

In addition to participant agreement, another indicator of codability is the existence of dedicated lexical resources, i.e., codable domains tend to have elaborate domain-specific terminology^3,38^. So, what are the Maniq and Polish lexicons for facial movements like? To explore this, we looked at all the terms employed to describe facial movements in both languages.

We found that Maniq had more domain-specific terms for facial movements compared to Polish. Polish speakers used predominantly general-purpose verbs such as *ruszać* ‘to move’, *marszczyć* ‘to wrinkle’, *podnosić/unosić* ‘to raise’ (and corresponding verb-derived nouns, e.g., *zmarszczenie* ‘wrinkling’), etc. For Maniq, by far the predominant strategy was the use of specialized verbs referring to facial movements, e.g., *kiɲus* ‘to wrinkle nose’, *biʔuɲ* ‘to open eyes widely’, and *yikieh* ‘to part lips’. The total number of such verbs recorded in Maniq across both tasks was 27. While domain-specific verbs were present also in Polish, e.g., *mrugać* ‘to blink’, *mrużyć* ‘to squint’, they were much less numerous than in Maniq—only 4 such verbs were recorded (for the full list for both languages, see Supplementary Tables S8 and S9).

Domain-specific verbs for facial movements refer to movements in concrete facial areas. For instance, the Maniq verb for raising eyebrows—*lɨt*—refers uniquely to the movement of this face part and may not be used with similar actions elsewhere on the face or in other contexts. Most of the Maniq facial action verbs can be mapped onto one or more action units, covering a broad range of facial movements associated with emotional expressions, as well as various behavioral and communicative functions. For example, they include face signals evolved for an enhanced rapid reaction to threats, e.g., *biʔuɲ* ‘to open eyes widely’ (AU5 “upper lid raiser”), *kiɲus* ‘to wrinkle nose’ (AU9 “nose wrinkler”) (cf. ref.^73,74^), movements that comprise complex emotional expressions, as well as those that are commonly used as communicative signals in face-to-face conversation, e.g., *lɨt* ‘to raise eyebrows’ (AU1, AU2 “outer/inner brow raiser”), *citũn* ‘to raise upper lip’ (AU10 “upper lip raiser”), and *klatis* ‘to turn mouth corners down” (AU15 “lip corner depressor) (cf. ref.^75,76^).

**Fig. 3:**
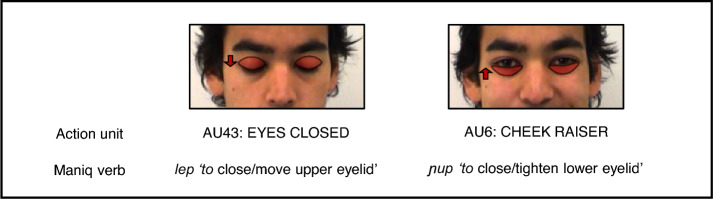
Eye-closing movements and their descriptions in Maniq. Two types of eye-closing movements, their FACS codes, and Maniq verbs used to describe them. Adapted from the Amsterdam Dynamic Facial Expression Set^60^ (reproduced with permission).

What is special about these verbs are their remarkably detailed meanings, with fine aspects of facial expressions coded lexically. For example, two distinct verbs exist for eye-closing movements in Maniq, depending on whether upper or lower eyelids are more visually prominent in the movement: the verb *lep* is used with upper eyelids (as in e.g., AU41 “lid droop”, AU43 “eyes closed”, and AU45 “blink”) (Fig. 3, left) and the verb *ɲup* with lower eyelids (as in e.g., AU7 “lid tightener” and AU6 “cheek raiser”) (Fig. 3, right) (note that for both verbs the movement can involve closing eyes either fully or partially, and momentarily or for a longer time).

In summary, Maniq and Polish differ significantly in the lexical resources applied to describe facial movements. While Polish relies primarily on general-purpose vocabulary, Maniq has a large lexicon of verbs dedicated to facial movements. These verbs have precise meanings referring to movements of specific parts of the face and are used with relatively high consensus. The high codability reported in the previous section is thus accompanied by considerable lexical specialization for facial movements in Maniq.

### Dedicated lexicon for mental states and the linguistic categorization of facial expressions of emotion

**Fig. 4:**
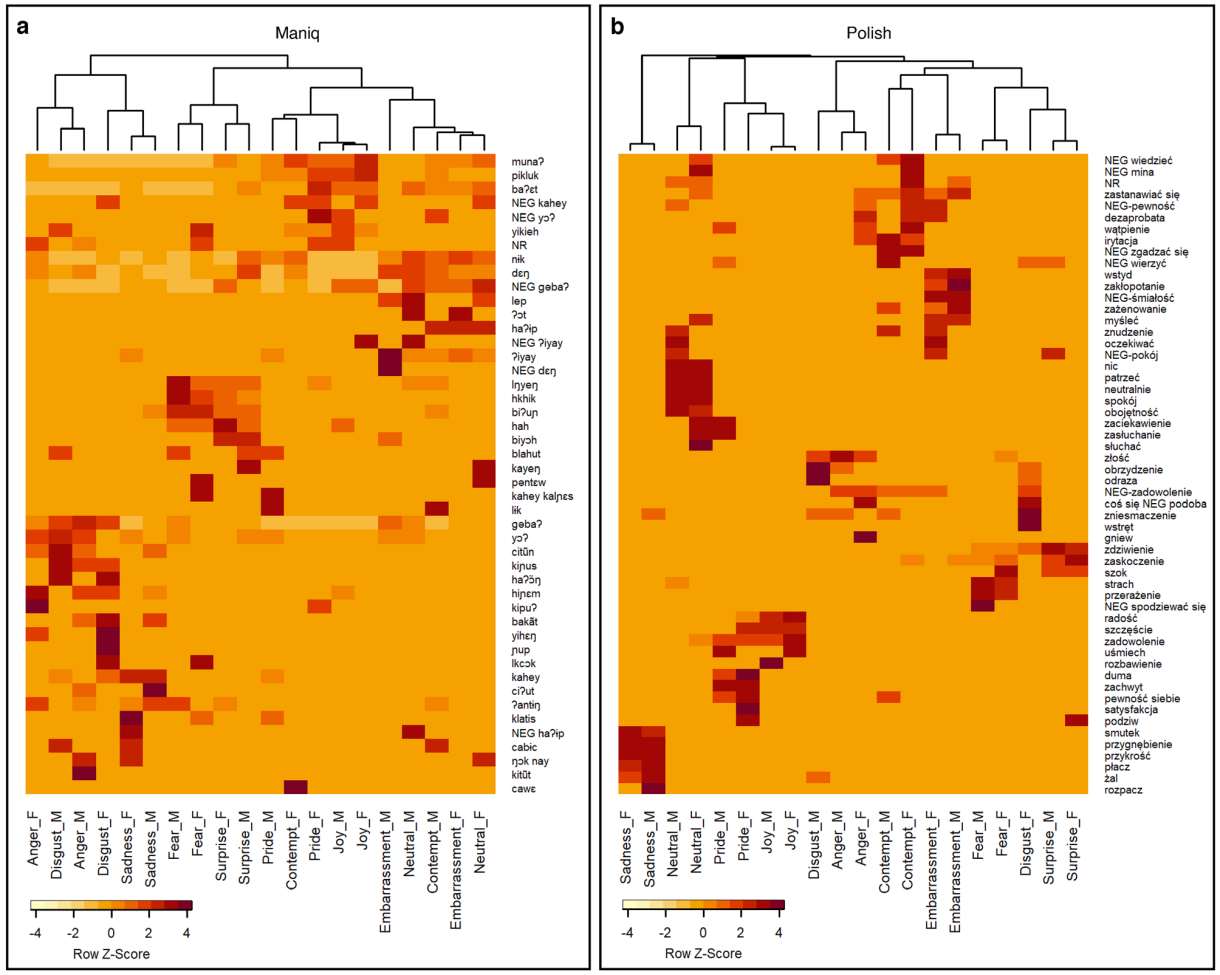
Co-occurrence matrices and dendrograms illustrating emotion stimuli groupings based on the naming data in Maniq and Polish. The values displayed in the co-occurrence matrices are scaled by rows. On each dendrogram, the length of branches represents similarity, i.e., the shorter the branches the more similar the stimuli. The dendrograms were calculated with an average-link agglomerative clustering algorithm using cosine distances. The letter F in the stimulus name stands for ‘female’ and M for ‘male’ (actor). For translations of the Maniq and Polish responses, see Supplementary Tables S12 and S13.

To complete the picture, we next examined the second lexical area of interest—the mental state lexicon. Since our results showed a stark contrast in the frequency of use of mental state vocabulary between Maniq and Polish speakers, we wanted to see whether this disproportion would also be associated with qualitative differences in mental state vocabulary. To explore this, we performed a cluster analysis, in which the naming data from the emotion task was used as input (for details, see Statistical analyses in the “Methods” section). Figure 4 contains co-occurrence matrices, for both Maniq and Polish, constructed on the basis of how often each term (considering terms used more than once) was used to describe each facial expression. The dendrograms, derived from underlying stimulus-by-stimulus similarities, represent the linguistic categorization of facial expressions of emotion for each of the two languages.

Comparing co-occurrence matrices in Fig. 4, Maniq responses were generally applied to a broader range of different facial expressions than Polish responses, which were more specialized, as indicated by the more well-delineated boundaries. This reflects the greater use of bodily action descriptors in Maniq and the lower semantic specificity (granularity) of Maniq mental state terms. For instance, for sad feelings Maniq has a single abstract emotion term *ʔiyay*, which in some contexts translates as ‘sad’, but it can also mean ‘upset’ or ‘irritated’, whereas in Polish there are multiple different ‘sad’ terms with more specific meanings—*smutek* ‘sadness’*, przygnębienie* ‘dejection’*, przykrość* ‘hurt’*, żal* ‘regret’*, rozpacz* ‘despair’ (see also the full list of mental state responses in Supplementary Tables S10 and S11). In line with this, clusters in the Maniq dendrogram are less distinct, with shorter distances between subclusters, compared to Polish.

In Polish, the clusters largely correspond to the emotion categories targeted by the stimuli (sadness, neutral, pride, joy, anger, contempt, embarrassment, fear, surprise). The exception is disgust, which did not form a distinct cluster. Instead, the two disgust stimuli fell into separate groupings: one with anger and one with surprise. In Maniq, there are four major clusters (from the left): (1) anger/disgust/sadness; (2) fear/surprise; (3) pride/contempt/joy; and (4) embarrassment/neutral/contempt. The pairing of anger and disgust as well as fear and surprise represent well-attested co-occurrences^23,77^. Pride, joy, and one instance of contempt represent positively valenced expressions, whereas embarrassment, the neutral expression, and the second instance of contempt are associated with lack of negative feelings or emotional content. For each cluster, the responses include also terms corresponding to some characteristic action units shared by the relevant expressions, e.g., (1) AU9 “nose wrinkler”, *kiɲus* ‘to wrinkle nose’; (2) AU5 “upper lid raiser”, *biʔuɲ* ‘to open eyes widely’; (3) AU25 “lips part”, *yikieh* ‘to part lips’; and (4) AU45 “blink”, *lep* ‘to close or move upper eyelid’.

Overall, while the linguistic categorization of emotion stimuli in Polish, except for disgust, seems to correspond to the proposed universal basic emotion categories, the Maniq data does not lend similar support to the existence of the proposed universal expressions of emotion^72,78–80^. This is especially true of contempt and disgust, with disparate affiliations for each instance of these emotions, questioning their status as distinct emotional signals. Our data is more consistent with alternative theories of emotion, where facial expressions corresponding to emotions such as disgust, anger, contempt, etc., are not discrete universal categories, but belong to larger expressive patterns conveying more general emotional information^77,81^. However, given the preliminary nature of this evidence and the fact that the linguistic structure of a domain does not always correspond to the conceptual structure^82^, for more definitive conclusions, further research with Maniq speakers is needed employing different task paradigms, e.g., pile sorting, choice-from-array with emotional scenarios, as conducted with other small-scale societies^24,23, 25^.

Most importantly for the present study, however, the cluster analysis illustrates qualitative differences between Maniq and Polish lexicons for facial expressions of emotion. Thus, not only do Polish speakers use mental state terms more frequently as shown in Fig. 2A, but the mental state lexicon in Polish is richer and more precise. The Maniq mental state lexicon, on the other hand, does not offer similar precision, naming emotions at a fairly general level and lacking dedicated abstract labels for many of the categories targeted in the stimuli. On the whole, the specificity of meanings, for Polish in the mental state domain, and for Maniq in the facial movement domain, shows these two languages have different foci of linguistic elaboration.

## Discussion

In this article, we asked how expressive faces are coded in different languages. Contrary to the idea that people tend to describe faces from the perspective of emotion, a cross-linguistic comparison between Maniq and Polish shows cultures in fact differ in this respect. Polish speakers reveal a tendency to use mentalistic descriptions of faces and attend to mental states. Maniq speakers, on the other hand, show a tendency to speak about faces in behavioral terms, paying special attention to facial movements. Crucially, they describe facial movements with ease, outperforming Polish speakers in codability, using an elaborate lexicon of dedicated verbs. Thus, even though details of faces are generally considered difficult to put into words, when it comes to facial movements, this is not universally true of all languages.

Such a result in Maniq might seem counterintuitive. If facial expressions evolved primarily to communicate mental states^12,13^, we would expect languages to be attuned to naming emotions rather than facial movements. Yet, as mentioned in the introduction, basic concepts of mental life, including concepts of emotions are culturally variable and can be differentially constituted, an observation backed by an exceptionally wide array of research, including work in psychology^22,83^, linguistics^17,21^, anthropology^27^, and clinical settings^26^. For Maniq speakers, action and bodily phenomenology are prominent aspects of the emotional experience, and action terms are foregrounded in Maniq speakers’ emotion descriptions (cf. similar observations for the Hadza^22^). For Polish speakers, on the other hand, bodily actions are secondary to mental states, and they do not tend to be foregrounded in descriptions of emotion to a similar degree. The differences in types of responses between Maniq and Polish speakers are thus largely a reflection of differences in emotion concepts across these two cultures.

Maniq and Polish also differ with the very makeup of the lexicons available in the two languages. While the Polish lexicon is tailored to mental states, as expected based on prior research^7,43^ and similar to English and many other large languages of industrialized societies, the Maniq lexicon is tailored to facial movements. This type of lexical specialization has not been reported before. The general consensus about the expressibility of physical aspects of faces is that although certain individual features can be named, facial descriptions tend to be imprecise as they “fail to capture the subtleties” of human faces^5^. So, facial lexicons are generally expected to be limited and not adapted to encoding the details of facial expressions. However, as demonstrated here, this is culturally variable and is best thought of as a continuum. Different languages offer different levels of specificity in basic vocabulary for facial movements, with Maniq revealing a remarkably rich and specific lexicon.

What factors lie behind such an outcome for Maniq? It is notable that the highly specialized lexicon for facial movements co-occurs with a much less rich and less differentiated mental state lexicon. These linguistic differences reflect the relatively low cultural salience of mental states compared to actions in the Maniq society and their limited role in the context of emotion (for a discussion of the ethnographic background shedding light onto this issue, see Supplementary Information). Thus, despite the relatively modest sample size of 13 participants per group, our quantitative results align with qualitative observations from the lexicon and are congruent with cultural observations, altogether presenting a coherent picture.

Our study raises interesting puzzles for face cognition. We know from previous work that language can modulate the processing of emotional faces. Importantly, different types of linguistic labels appear to do that in different ways. Using feature descriptors of faces has been shown to *interfere* with the perception of emotional configurations^53,84^, whereas using emotion labels can *enhance* such perception^54,55^. This prompts the question whether the linguistic differences reported here would be reflected in divergent cognitive patterns among speakers of different languages. Prior research in other domains (e.g., spatial relations) has demonstrated linguistic categories can be powerful guides to perception and can affect habitual attention patterns, for instance by sensitizing speakers to the aspects of reality coded in their language^85–87^ (although see ref.^88^). Similar variation might exist for expressive faces. In fact, perceptual modulations driven by language would not be unexpected, as holistic processing of faces has been argued to be modifiable and shaped by experience^65^. Dedicated research is necessary to assess this possibility, examining closely the potential robustness of any such effects and their exact relationship to language.

## Conclusion

Diverse languages exhibit systematic differences in the coding of facial expressions. Such a result would not have been predicted by Darwin or Wittgenstein, who stressed we are oriented to mental states on the face and unable to describe physical aspects of facial expressions. Yet, it is consistent with what one would expect given the variation in the cultural salience of mental states. People across cultures differ with how much they attend to mental states and what aspects of emotional experience they focus on. Here we contribute to the cumulative multidisciplinary effort to document this variation and for the first time demonstrate it is also carved into language. As illustrated with the Maniq data, details of facial expressions can be richly coded, surpassing mental states. All in all, how facial expressions are described in language does not adhere to a single universal blueprint but is malleable and can be shaped by culture.

## Methods

The research was approved by the National Research Council of Thailand and the Rector's Committee for the Ethics of Research Involving Human Participants at the University of Warsaw. The research reported in this article was conducted in accordance with their guidelines and regulations and complies with the ethical standards in the American Anthropological Association Code of Ethics. Polish speakers provided written informed consent, whereas Maniq speakers provided informed consent orally. Participant received compensation for participation amounting to approximately 7–8 USD (30 PLN, 250 THB), paid in cash (Polish group) or in requested food items (Maniq group). See Supplementary Information for a more detailed profile of the study populations and languages.

### Participants

The data was collected from 26 participants: 13 native Maniq speakers (6 female), and 13 native Polish speakers (6 female). As there are documented age effects in face recognition^89^ and facial expression processing^90^, we recruited participants within broadly similar age range (Maniq: range 18–65, *M* = 37, *SD* = 12; Polish: range 22–60, *M* = 37, *SD* = 12), so as to minimize the likelihood that any potential effects would be due to participants’ age. Given that the Maniq community is estimated to number 300 individuals living in small, mostly nomadic, groups^39^, recruiting a large number of participants is challenging. We recruited all willing-to-participate adult members of the Maniq group we had access to, resulting in 13 participants, and a matching number of Polish speakers. Maniq participants had no formal education, except for three people, who attended a school. Polish participants had secondary (non-university) education, except for two participants, who had university degrees. Maniq participants were recruited on site and Polish participants via internet, through advertisements on dedicated social media sites.

### Stimuli

For both experimental tasks, stimuli were short video clips presented to the participants on a laptop. The stimuli for the facial movement task consisted of 42 brief video clips from the iMotions website (https://imotions.com/blog/learning/research-fundamentals/facial-action-coding-system/), appearing in the center of the screen against black background, each displayed in separate browser tabs. Most facial action clips depict one action unit (AU)^59^, except for a small number of clips in which the targeted AU is typically accompanied by another one (e.g., nose wrinkler and upper lip raiser). The AUs were presented in the context of a part of a face, usually the upper or lower half, or the whole face for AUs depicting head movements. The stimuli for the emotion task consisted of 20 video clips selected from the Amsterdam Dynamic Facial Expression Set (ADFES)^60^. They depicted complex facial expressions corresponding to a set of specific emotions: surprise, disgust, anger, sadness, joy, fear, pride, embarrassment, and contempt, as well as a neutral expression. Dynamic stimuli were selected because they enhance emotion recognition and are associated with higher ecological validity compared to photos, as real-life expressions of emotion involve action^60^. There were two instances of each emotion, one with a female, and one with a male actor. Of the two ethnicities in ADFES—Northern European and Mediterranean—we selected Mediterranean actors, deemed less likely to be associated with an own-ethnicity (or own-race) advantage^91^ for either group. Critically, this choice was not predicted to be associated with processing-related differences, as both own-race and other-race facial expressions have been shown to rely on holistic processing^92^, and would not result in a greater likelihood to be interpreted in terms of actions. Each video clip showed the whole face seen from the front. The full list of stimuli used in the study is provided in Supplementary Tables S1 and S2.

### Procedure

Participants provided general demographic information (age, gender, education) and completed two experimental tasks. The order of the tasks was counterbalanced across participants. During each task, participants were shown a series of video clips in a fixed order and for each clip answered the question: “What is he/she doing?” (Polish: *Co on/ona robi?*; Maniq: *ʔɛʔ diʔ kaləw?*) in the facial movement task, and “What does he/she feel?” (Polish: *Co on/ona czuje?*; Maniq: *ʔɛʔ nɨk/rusɨk kaləw?*) in the emotion task. There was no time limit and participants were free to rewatch the clips. Their answers were audio-recorded and transcribed for later analysis. It took approximately 15–30 min to complete the two tasks.

### Statistical analyses

The following statistical analyses were performed on the data. All analyses were carried out in R.

#### Types of responses

We assessed whether the differences in response patterns ("bodily action" vs. "mental state" vs. “other”) across the tasks and languages were significant. To do that, we performed a series of Pearson’s Chi-squared tests for homogeneity, which checks whether the by-language and by-task pairs of observed distributions of types of responses originate from common distributions.

#### Codability of expressive faces

We applied linear mixed-effects modeling to determine whether stimulus codability, operationalized as Simson’s Diversity Index, differed significantly across languages and tasks. The method allows for modelling dependencies between variables while accounting for any expected random effects, such as the selection of stimuli. We tested main and simple effects of independent variables with likelihood ratio tests and t-tests on fitted coefficients, respectively.

#### Labeling consistency

We examined the consistency in labeling the basic emotion stimuli with expected emotion words. To see whether labeling consistency differed between Polish and Maniq speakers, we performed the Mann–Whitney U-test on the 24 observed proportions, one for each of the 12 stimuli in each of the two languages.

#### Cluster analysis of facial expression stimuli

We conducted a cluster analysis to examine linguistic categorization of facial expressions of emotion in Maniq and Polish. We generated co-occurrence matrices containing information on how often each term was used to describe each facial expression, considering terms used more than once. This information was then used to calculate cosine similarity with the cosine function from the lsa R package^93^, resulting in stimulus-by-stimulus similarity matrices for each language. Similarity matrices served as input into agglomerative hierarchical clustering, utilizing an average-link clustering method. The resulting emotion stimuli groupings are displayed in Fig. 4.

## Supplementary Information

Supplementary Information.

## Data Availability

The datasets generated and analyzed during the current studies are available in The Open Science Framework repository, at https://osf.io/aqkrs/?view_only=5aaebd0a92a94af588483024c84bea6d.
