# Supplementary material for: Culture shapes how we describe facial expressions
Source: Sci Rep. 2024 Sep 16;14:21589. doi: 10.1038/s41598-024-72432-w (PMC11405906; doi:10.1038/s41598-024-72432-w)
Supplement: Supplementary file 1 — Supplementary Information. [file 41598_2024_72432_MOESM1_ESM.pdf]

Supplementary Information for

Culture shapes how we describe facial expressions

Ewelina Wnuk[1]* & Jan Wodowski[1]

[1]Faculty of Modern Languages, University of Warsaw, Warsaw, 00-312, Poland

*Corresponding author: Ewelina Wnuk, em.wnuk2@uw.edu.pl

This file includes:

1. General description of the study populations
2. Response coding procedure
3. Ethnographic background to mental states and actions in the Maniq society
4. Supplementary Tables S1-S2: Information about the stimuli used in the experimental tasks
5. Supplementary Table S3: Frequencies of different types of responses across tasks and languages
6. Supplementary Tables S4-S5: Summary descriptions of the mixed-effect models
7. Supplementary Table S6: Summary statistics of the codability measures across tasks and languages
8. Supplementary Table S7: Consistency of labeling with the expected emotion terms
9. Supplementary Table S8-S9: Lists of all elicited domain-specific terms for facial movements in Maniq and Polish
10. Supplementary Table S10-S11: Lists of all elicited expressions categorized as mental states in Maniq and Polish
11. Supplementary Table S12-S13: Translations of all Maniq and Polish responses used in the cluster analysis
12. References

1. General description of the study populations

1.1. Maniq

The Maniq are a traditional hunter-gatherer group living in a tropical rainforest in Southern Thailand. The population numbers about 300 members, living in groups of an average size of 25-35 people. Most Maniq are nomadic, with some variation from group to group[1]. The group composition and residential unit size vary across the year. The Maniq inhabit the area of the Banthad mountain range stretching across four southern provinces of Thailand (Trang, Satun, Phatthalung, and Songkhla). The group participating in this study is from the area of the Manang district, Satun province. The Maniq society is organized according to egalitarian social norms and practices such as extensive sharing and social taboos against status seeking behaviors[2].

Maniq is an unwritten language of the Austroasiatic language family, the Aslian branch. Its formal properties include a rich phonemic inventory, intricate derivational morphology, frequent argument ellipsis, and semantically dense verb lexicon with highly specific meanings[1]. All Maniq speakers are also good or fluent speakers of Southern Thai, used in interactions with outsiders. Maniq is the primary language of everyday communication and the first language learnt by children.

1.2. Polish

There is an estimated number of 40 to 45 million speakers of Polish in the world, of which about 38 million in Poland itself[3]. Most Poles live in large urban areas and like other large, industrialized nations are exposed to modern technology, globalized media, and popular culture. Participants in this study were inhabitants of Warsaw, the Polish capital, and were speakers of a standard variety of Polish.

Polish is a Slavic language of the Indo-European language family. Unlike Maniq, it has a long history of written texts and a strong prescriptivist tradition in orthography and grammar. It contains borrowed vocabulary from other Slavic, Romance, and Germanic languages, including many lexical items which are cognates of modern English, French, and German words (such as the mental state terms *ekscytacja* ‘excitement’, *satysfakcja* ‘satisfaction’, *irytacja* ‘irritation’). About two thirds of adult Polish speakers (age 18-69) also speak a foreign language, most commonly English, with levels of proficiency varying from basic, intermediate, to advanced[4].

2. Response coding procedure

2.1. Main response identification

Similar to other cross-linguistic investigations of codability[5–9], coding was focused on individuating semantic heads of each response, rather than morpho-syntactically-defined categories. The procedure was the same for Maniq and Polish and was sensitive to the linguistic systems of both languages, considering their structural features and irregularities (see below).

Both studies involved free description, so participants could provide multiple responses. We took into account all responses provided, unless the participants explicitly signaled a correction. When someone indicated they were unable to describe a clip, e.g., by saying "I don't know", "nothing comes to mind", etc., we coded that as "NR" (no response). If a response was missing due to experimenter's error, this was coded as "NA" (non-applicable).

In our coding, we identified main responses, i.e., the elements carrying the semantic gist of the descriptions, which constituted further units of analysis. To do that, we removed any function words, grammatical morphemes, and modifiers, and extracted the common roots of formally related responses. We also omitted body part names since the key information we were interested in for the two languages was how easy it was to name the *actions* and *states* of the persons in the videos in each of them. Similarly, we disregarded those parts of the responses which did not describe the person in the video but elaborated on imagined background scenarios. A few examples illustrating the basic coding principles are provided below:

(1) *Myślę, że jest to forma jakiejś aprobaty* (Polish)
'I think it's a form of approval.'
Main responses: 1. *aprobata* 'approval'

(2) *Ruch brwi, zmarszczenie brwi, no i tyle* (Polish)
'Eyebrow movement, eyebrow wrinkling, and that's it.'
Main responses: 1. *ruch* 'movement' 2. *zmarszczenie* 'wrinkling'

(3) *ʔɛʔ limiŋyeŋ, mɛt hay dɔn* (Maniq)
'She's showing the whites of her eyes, her eyes are like that of a slow loris (Nycticebus coucang).'
Main responses: 1. *lŋyeŋ* 'to show the whites of one's eyes' 2. *hay dɔn* 'like slow loris'

(4) *ʔɛʔ ʔiyay ganaʔ, wa pãʔ ʔisiʔ* (Maniq)
'He's upset about his partner, she went off to another man.'
Main responses: 1. *ʔiyay* 'to be upset'

For responses involving complex predicates, where both verbs contributed lexical information, e.g., multi-verb constructions (Maniq) or converbal constructions (Polish), each verb from the predicate was included as a distinct response, e.g., in *pimikluk cimintũn*

‘smiling raising upper lip’ (Maniq), both the verb *pikluk* ‘to smile/laugh’ and *citũn* ‘to raise upper lip’ were main responses. For modal complement-taking predicates, e.g., *próbować ukryć* ‘try to hide something’ (Polish), *chcieć wydać dźwięk* ‘want to make a sound’ (Polish), the main response was the entire predicate.

Formally related responses which shared a common root were coded as the same category, e.g., the Polish noun *ekscytacja* ‘excitement’ and adjective *podekscytowany* ‘excited’. Similarly, morphologically complex verbs and nouns with shared roots and distinct prepositional prefixes were collapsed, but only if their meaning was highly similar, e.g., *pod-nosić* ‘to raise’ (under-carry) and *u-nosić* ‘to raise’ (at-carry), and not if there was a semantic difference between them, e.g., *przy-mykanie* ‘closing slightly’ and *za-mykanie* ‘closing entirely’. If words sharing a common root differed in their semantics, however, we treated them as distinct. For example, in some cases two or more words were formally related, but represented only partially overlapping concepts, e.g., the Polish adjective *zła* ‘angry/bad/evil’ and noun *złość* ‘anger’. Such cases were regarded as separate responses. Finally, we collapsed some morphologically unrelated, but semantically paired elements such as the perfective and imperfective verb roots in Polish, e.g., *mówić* ‘to say (imperfective)’ and *powiedzieć* ‘to say (perfective)’.

Some responses involved a negating element (word or morpheme), e.g., the Maniq phrase *hɨc/bah gəbaʔ* ‘is not angry’ (NEG be.angry). Because both Maniq and Polish use multiple negators, we coded negation as “NEG”, so as not to differentiate between different negating elements, e.g., *hɨc gəbaʔ* and *bah gəbaʔ* were both coded as NEG-*gəbaʔ*.

2.2. Response type classification

Once the main responses have been identified, we classified them according to response type. There were two main types of responses: “bodily actions” and “mental states”. A small number of responses which did not fit easily into either of these two categories were classified as “other”.

The bodily action category included descriptions of simple movements of facial muscles or the head, e.g., *marszczyć brwi* ‘to wrinkle one’s brows’ (Polish), *cikiey* ‘to turn one’s head sideways or back’ (Maniq), as well as descriptions of more complex physical actions, e.g., *śmiać się* ‘to laugh’ (Polish), *kahey* ‘to cry’ (Maniq), *słuchać* ‘to listen’ (Polish), and *biyɔh* ‘to speak’ (Maniq). Generally speaking, this category was associated with specific actions performed with the body, as opposed to more abstract actions (classified as “other”), e.g., *udawać* ‘to pretend’ (Polish), *przyciągać uwagę* ‘to draw attention’ (Polish), *przywitać się* ‘to greet’ (Polish), where bodily action was implied but the lexicalized meaning component concerned the actor’s intentions and goals.

The mental state category included descriptions of emotions, e.g., *wstyd* ‘shame’ (Polish), *munaʔ* ‘be happy, glad’ (Maniq), cognitive activity, e.g., *myśleć* ‘to think’ (Polish), *haʔɨp* ‘to know, to remember’ (Maniq), and expressions of will, e.g., *chcieć skończyć rozmowę* ‘to

want to end a conversation' (Polish).

Intermediate cases, whereby both mental and bodily aspects were involved, were classified consistent with their primary denotative meaning, e.g., *kahey* (Maniq)/*płakać* (Polish) 'cry' and *pikluk* (Maniq)/*śmiać się* (Polish) 'laugh' were coded as "bodily action", unless such bodily actions were used figuratively to express an inner feeling, e.g., *kahey kalɲɛs* 'to be crying inside one's heart' (Maniq) classified as mental state.

Finally, responses coded as "other" included a variety of descriptions not falling into either category. These included: abstract actions with lexicalized intention or goal, e.g., *udawać* 'to pretend' (Polish), physiological states and sensations, e.g., *campɨs* 'ill' (Maniq), perceived similarity to action or appearance of an animal, e.g., *hay bəwac* 'like a pig-tailed macaque' (Maniq), and general descriptions of appearance, e.g., *suche usta* 'dry lips' (Polish). Most of the responses classified as "other" were infrequent; in fact, the majority of them were used only a single time. The top three responses in this category were: *NR* '(no response)' (attested 26 times), *nic* 'nothing' (Polish) (attested 8 times), and *potwierdzać* 'to confirm' (Polish) (attested 6 times).

3. Ethnographic background to mental states and actions in the Maniq society

Observations from long-term ethnographic fieldwork with Maniq people suggest references to mental states are not a prominent part of everyday discourse. Though mental state terms such as *ʔantɨŋ* 'to be afraid' and *məwɛ̃ŋ* 'to feel lazy, reluctant' are common everyday words, it is rare for speakers to linger on descriptions of mental states or center conversations around them. Even in situations which seem particularly emotion-laden, people typically do not focus their narratives on mental states but tend to foreground external events. For instance, when in February 2014 one family's shelter caught fire while they were asleep and burnt down, members of this household related this story by highlighting key events and did not spontaneously reference mental states.

The low salience of mental states holds also in relation to other minds. Although the Maniq society does not seem to have an explicit opacity of mind doctrine similar to the one of Samoans[10], mental states of other people are typically not in focus in ordinary talk. In some contexts, e.g., when talking about strangers, it might be relevant to specify whether or not they are angry/aggressive (*gəbaʔ*), as threatening behavior by outsiders historically used to be, and sometimes still is, a concern for Maniq people[11]. The importance of this determination is reflected in the presence of the phrase *hɨc gəbaʔ* 'not angry/aggressive' in the emotion naming task, with faces ranging from happy, neutral, to embarrassed, all receiving this description from time to time (note that no similar response was recorded in the Polish data). Beyond this coarse-level distinction, however, spontaneous references to mental states of others are relatively infrequent. When casually viewing old photos of their own group, for instance, Maniq speakers tend to reference actions and other physical details of the scene and only rarely mention any mental states.

Thus, mental states do not tend to be focal in discourse, as reflected also in the Maniq lexicon for mental states being relatively small and semantically general. What cultural factors could explain this? Certainly, the Maniq society is not isolated in this phenomenon. Lexical mental state distinctions are similarly coarse-grained in at least some of the close linguistic relatives of Maniq, e.g., Ceq Wong of Malaysia[12]. It has been suggested talking openly about mental states of other people clashes with egalitarian values of personal autonomy, as it amounts to claiming undue authority over others, and might therefore be culturally constrained[13]. This could well be a relevant factor for Maniq people, who as egalitarian hunter-gatherers attach importance to respecting the bounds of personal autonomy. However, the low salience of mental states is a broader phenomenon in the Maniq society, not limited exclusively to other minds and is best viewed in the wider context of emotional events and the Maniq concept of a person.

Rich anthropological research shows that the way persons are conceptualized is not a universal, but differs across societies and is fundamentally important for how people might conceive of their mental life[14–16]. Crucially, the Maniq concept of a person has been described as “action-oriented”, meaning that Maniq people define personhood primarily on the basis of actions rather than objectified essence[17] (see also ref. [18]). For example, identifying someone as Maniq is critically dependent not on ethnicity, but on actions considered intrinsic to “Maniq-ness” such as hunting, gathering, and eating forest foods[19]. So, being able to call oneself Maniq necessarily requires engaging in these actions. In the context of emotion, similarly, considerable focus is placed on actions, i.e., what one does in the context of emotion-laden events. Private thoughts and feelings, on the other hand, are not foregrounded in the same way as they are in the Euro-American context[14]. For instance, when asked about their reaction to the loss of a loved one, e.g., a mother, Maniq people typically mention what they do, e.g., visit and spend time with their siblings, and do not volunteer information about internal mental states.

These observations are consistent with the linguistic data presented in the article. In the emotion task, Maniq speakers used action descriptions half of the time, even though the instruction targeted mental states, whereas Polish speakers used primarily dedicated emotion terminology. In addition, the Maniq mental state lexicon itself is oriented to actions and is distinctly “verby” in its grammatical properties[1]. While typical English translations of Maniq terms involve adjectives or nouns, e.g., ‘to be angry’, ‘to feel anger’, in Maniq by default they function as verbs and reveal a conceptualization akin to actions. Polish terms, on the other hand, are more flexible and depending on the specific concept can be coded as verbs, but they are also commonly expressed as nouns (unattested with mental state terns in Maniq).

Supplementary Table S1

Information about the stimuli - facial movement task			
**Order of presentation**	**Stimulus id**	**Action Unit number**	**Action Unit name**
1	201	1	Inner Brow Raiser
2	202	2	Outer Brow Raiser
3	203	4	Brow Lowerer
4	204	5	Upper Lid Raiser
5	205	6	Cheek Raiser
6	206	7	Lid Tightener
7	207	9	Nose Wrinkler
8	208	10	Upper Lip Raiser
9	209	11	Noasolabial Deepener
10	210	12	Lip Corner Puller
11	211	13	Cheek Puffer
12	212	14	Dimpler
13	213	15	Lip Corner Depressor
14	214	16	Lower Lip Depressor
15	215	17	Chin Raiser
16	216	18	Lip Puckerer
17	217	20	Lip Strecher
18	218	22	Lip Funneler
19	219	23	Lip Tightener
20	220	24	Lip Pressor
21	221	25	Lips Part
22	222	26	Jaw Drop
23	223	27	Mouth Stretch
24	224	28	Lips Suck
25	225	41	Lid Droop
26	226	42	Slit
27	227	43	Eyes Closed
28	228	44	Squint
29	229	45	Blink
30	230	46	Wink
31	231	51	Head Turn Left
32	232	52	Head Turn Right
33	233	53	Head Up
34	234	54	Head Down
35	235	55	Head Tilt Left
36	236	56	Head Tilt Right
37	237	57	Head Forward
38	238	58	Head Back
39	239	61	Eyes Turn Left
40	240	62	Eyes Turn Right
41	241	63	Eyes Up
42	242	64	Eyes Down

Supplementary Table S2

Information about the stimuli - emotion task			
**Order of presentation**	**Stimulus id**	**Target emotion**	**File names from Amsterdam Dynamic Facial Expression Set**
1	101	Embarrassment	F09-Embarrass-Face Forward.mpeg
2	102	Contempt	M01-Contempt-Face Forward.mpeg
3	103	Neutral	M01-Neutral-Face Forward.mpeg
4	104	Surprise	M01-Surprise-Face Forward.mpeg
5	105	Disgust	F09-Disgust-Face Forward.mpeg
6	106	Neutral	F09-Neutral-Face Forward.mpeg
7	107	Anger	M01-Anger-Face Forward.mpeg
8	108	Sadness	F09-Sadness-Face Forward.mpeg
9	109	Embarrassment	M01-Embarrass-Face Forward.mpeg
10	110	Disgust	M01-Disgust-Face Forward.mpeg
11	111	Joy	F09-Joy-Face Forward.mpeg
12	112	Surprise	F09-Surprise-Face Forward.mpeg
13	113	Fear	M01-Fear-Face Forward.mpeg
14	114	Pride	M01-Pride-Face Forward.mpeg
15	115	Contempt	F09-Contempt-Face Forward.mpeg
16	116	Sadness	M01-Sadness-Face Forward.mpeg
17	117	Fear	F09-Fear-Face Forward.mpeg
18	118	Anger	F09-Anger-Face Forward.mpeg
19	119	Pride	F09-Pride-Face Forward.mpeg
20	120	Joy	M01-Joy-Face Forward.mpeg

Note: The letter F in the file name stands for ‘female’ and M for ‘male’ (actor).

Supplementary Table S3

Types of responses<br>Summary of the frequencies of different types of responses across languages and tasks		
	**N**	**Fraction**
Maniq - Emotion		
bodily action	276	51.493%
mental state	242	45.149%
other	18	3.358%
Total	536	100%
Maniq - Facial movement		
bodily action	749	94.930%
mental state	4	0.507%
other	36	4.563%
Total	789	100%
Polish - Emotion		
bodily action	24	5.714%
mental state	373	88.810%
other	23	5.476%
Total	420	100%
Polish - Facial movement		
bodily action	513	68.952%
mental state	171	22.984%
other	60	8.065%
Total	744	100%

Supplementary Table S4

Model 1 Summary

Model equation: logSDI ~ Language * Task + (1| Stimulus_id)

Fixed effects

	B	SE	95% CI(B) lower[1]	95% CI(B) higher[1]	Df[2]	t[2]	p[2]
(Intercept)	–1.595	0.099	–1.789	–1.401	110.775	–16.105	0.000
LanguagePolish	–0.907	0.118	–1.138	–0.675	60.000	–7.678	0.000
TaskEmotion	–0.702	0.174	–1.044	–0.361	110.775	–4.029	0.000
LanguagePolish:TaskEmotion	0.933	0.208	0.525	1.341	60.000	4.486	0.000

[1] calculated using the Wald method
[2] calculated using Satterthwaite's approximations

Random effects

Groups	Name	Var	SD
Stimulus_id	(Intercept)	0.119	0.345
Residual	NA	0.293	0.541

Model fit

Log-likelihood
–121.174

Supplementary Table S5

Model 2 Summary

Model equation: logSDI ~ Language * Task + (1| Stimulus_id)
Predictor reference values: Language: Polish; Task: Emotion

Fixed effects

	B	SE	95% CI(B) lower[1]	95% CI(B) higher[1]	Df[2]	t[2]	p[2]
(Intercept)	−2.271	0.144	−2.553	−1.990	110.775	−15.828	0.000
relevel(Language, ref = "Polish")Maniq	−0.026	0.171	−0.361	0.310	60.000	−0.152	0.880
relevel(Task, ref = "Emotion")Facial movement	−0.230	0.174	−0.572	0.111	110.775	−1.322	0.189
relevel(Language, ref = "Polish")Maniq:relevel(Task, ref = "Emotion")Facial movement	0.933	0.208	0.525	1.341	60.000	4.486	0.000

[1] calculated using the Wald method
[2] calculated using Satterthwaite's approximations

Random effects

Groups	Name	Var	SD
Stimulus_id	(Intercept)	0.119	0.345
Residual	NA	0.293	0.541

Model fit

Log-likelihood
−121.174

Supplementary Table S6

Codability of facial movement

Summary statistics for codability, Simpson's Diversity Index (SDI), across the languages and tasks

	SDI		logSDI[1]			
	M(SDI)	SD(SDI)	M(logSDI)	SD(logSDI)	95% CI(logSDI) lower	95% CI(logSDI) higher
Maniq						
Facial movement	0.234	0.170	−1.595	0.595	−1.780	−1.409
Emotion	0.102	0.056	−2.297	0.472	−2.518	−2.076
Polish						
Facial movement	0.090	0.075	−2.502	0.621	−2.695	−2.308
Emotion	0.134	0.112	−2.271	0.885	−2.685	−1.857

[1] SDI normalized with function: logSDI = log(SDI + 0.01)

Supplementary Table S7

Emotion labeling consistency

Emotion labeling consistency with the expected emotion terms across the stimuli and languages

	Target emotion	Labeling consistency with the expected term	SD	95% Agresti-Coull CI lower	95% Agresti-Coull CI higher
Polish					
101	Embarrassment	0.462	0.499	0.232	0.709
109	Embarrassment	0.692	0.462	0.420	0.876
102	Contempt	0.385	0.487	0.176	0.646
115	Contempt	0.077	0.266	−0.007	0.354
104	Surprise	1.000	0.000	0.734	1.038
112	Surprise	1.000	0.000	0.734	1.038
105	Disgust	0.538	0.499	0.291	0.768
110	Disgust	0.615	0.487	0.354	0.824
107	Anger	0.692	0.462	0.420	0.876
118	Anger	0.615	0.487	0.354	0.824
108	Sadness	0.846	0.361	0.565	0.969
116	Sadness	0.769	0.421	0.491	0.925
111	Joy	1.000	0.000	0.734	1.038
120	Joy	0.923	0.266	0.646	1.007
113	Fear	0.769	0.421	0.491	0.925
117	Fear	0.538	0.499	0.291	0.768
114	Pride	0.385	0.487	0.176	0.646
119	Pride	0.308	0.462	0.124	0.580
Maniq					
101	Embarrassment	0.000	0.000	−0.038	0.266
109	Embarrassment	0.000	0.000	−0.038	0.266
102	Contempt	0.000	0.000	−0.038	0.266
115	Contempt	0.000	0.000	−0.038	0.266
104	Surprise	0.000	0.000	−0.038	0.266
112	Surprise	0.000	0.000	−0.038	0.266
105	Disgust	0.000	0.000	−0.038	0.266
110	Disgust	0.000	0.000	−0.038	0.266
107	Anger	0.769	0.421	0.491	0.925
118	Anger	0.385	0.487	0.176	0.646
108	Sadness	0.154	0.361	0.031	0.435
116	Sadness	0.077	0.266	−0.007	0.354
111	Joy	0.923	0.266	0.646	1.007
120	Joy	0.769	0.421	0.491	0.925
113	Fear	0.154	0.361	0.031	0.435
117	Fear	0.000	0.000	−0.038	0.266
114	Pride	0.000	0.000	−0.038	0.266
119	Pride	0.000	0.000	−0.038	0.266

Note: Past facial configuration naming studies used various approaches to determining which terms should be counted as expected and considered accurate[19, 20]. In this analysis, we focus exclusively on emotion terms proper (we are not taking into account literal descriptions of bodily actions). The expected emotion terms used in the task were: (1) joy: Maniq - *baʔɛt* ‘be good, happy’, *munaʔ* ‘be happy, glad’; Polish - *radość* ‘happiness’, *szczęście* ‘joy’, *zadowolenie* ‘contentment’; (2) sadness: *ʔiyay* ‘be sad, irritated, upset’, *bakãt kalɲɛ*s ‘be crying in one’s heart’; Polish - *boleść* ‘pain, hurt’, *przygębienie* ‘dejection’, *przykrość* ‘unpleasantness, hurt’, *rozżalenie* ‘regret, sorrow’, *smutek* ‘sadness’, *załamanie* ‘devastation’, *żal* ‘regret, sorrow’, *rozpacz* ‘despair’; (3) anger: Maniq - *gəbaʔ* ‘be angry’, *yɔʔ* ‘be angry’; Polish - *wrogość* ‘hostility’, *złość* ‘anger’, *gniew* ‘anger’, *irytacja* ‘irritation’, *naburmuszona* ‘grumpy’; (4) fear: Maniq - *ʔantɨŋ* ‘be afraid’; Polish - *lęk* ‘anxiety’, *przerażenie* ‘terror’, *strach* ‘fear’; (5) surprise: no dedicated emotion term in Maniq; Polish - *szok* ‘shock’, *zaskoczenie* ‘surprise’, *zdziwienie* ‘surprise, bewilderment’; (6) disgust: no dedicated emotion term in Maniq; Polish - *odraza* ‘disgust’, *wstręt* ‘disgust’, *zniesmaczenie* ‘distaste’, *obrzydzenie* ‘disgust’, *ohyda* ‘repulsion’. Note that in Maniq there are no dedicated emotion terms for surprise and disgust. Thus, for a more nuanced comparison it is instructive to consider individual stimuli.

Supplementary Table S8

Maniq: Domain-specific terms for facial movements<br>A list of all unique Maniq responses dedicated to facial movements used across the two tasks	
**Maniq description**	**English translation**
bilay	to move one's eyes up, to look up
biʔuɲ	to open one's eyes widely
cakip	to bow one's head sharply
cikiey	to turn one's head sideways or back, to look sideways or back
citũn	to raise upper lip
ciyɛ̃k	to move one’s eyes to the side, to look sideways
hah	to open one's mouth
hapit	to close one's mouth
haʔɛ̃m	to squint
hiɲɛm	to squint
hlabit	to close one's eyes
kiɲus	to wrinkle nose
kipĩẽh	to narrow one's nostrils
klatis	to turn mouth corners down
lep	to close or move upper eyelid
lɨt	to raise eyebrows
mlyel	to move tongue
ɲup	to close or tighten lower eyelid
ŋap	to make movements with one's mouth like when speaking or eating slowly
pɨh	to open one's eyes
wɔt	to tilt one's head to the side
yɔp	to move one's eyes down, to look down
yihɛŋ	to widen one's nostrils
yikieh	to part lips, usually also showing teeth
yikũɲ	to pucker lips, to put lips in a kissing/whistling shape
yiwɛŋ	to pull lip corners as when smiling without showing teeth, to make one's dimples show
yok	to move one's head forward (similar to the horizontal head movement characteristic of chickens)

Supplementary Table S9

Polish: Domain-specific terms for facial movements

A list of all unique Polish responses dedicated to facial movements used across the two tasks

Polish description	English translation
mrugać	to blink
mrużyć	to squint
szczerzyć	to grin
wytrzeszczać	to goggle

Supplementary Table S10

Maniq: Mental state responses	
A list of all unique Maniq responses categorized as mental states used across the two tasks	
**Maniq description**	**English translation**
ʔiyay	to be sad, irritated, upset
munaʔ	to be happy
gəbaʔ	to be angry
yɔʔ	to be angry
baʔɛt	to be good, happy
ʔantɨŋ	to be afraid
jijɛy	to be happy, glad (Thai loan)
ʔɔt	to miss, to lack
nɨk	to think, to feel
NEG gəbaʔ	to not be angry
NEG ʔantɨŋ	to not be afraid
NEG ʔiyay	to not be sad, irritated, upset
NEG haʔɨp	to not know, to not remember
NEG maʔo	to not be unpleasant
NEG yɔʔ	to not be angry
bakãt kalɲɛs	to be crying inside ('in one's heart')
haʔɨp	to know, to remember
kahey kalɲɛs	to be crying inside ('in one's heart')

Supplementary Table S11

Polish: Mental state responses

A list of all unique Polish responses categorized as mental states used across the two tasks

Polish description	English translation	Polish description	English translation
akceptacja	acceptance	opór	resistance
analizować	to analyze	pewność siebie	self-confidence
antypatia	antipathy	podoba się	[something] is to [someone's] liking
aprobata	approval	podstęp	deceit
bać się	to fear	podziw	admiration
boleść	pain, hurt	próbować ukryć	to try to hide [something]
być w innym świecie	to be in another world	przerażenie	terror
chcieć komuś coś zaakcentować	to want to emphasize something to someone	przeskrobać coś	to make mischief
chcieć poderwać	to want to pick up	przygnębienie	dejection
chcieć powiedzieć	to want to say [something]	przyjemność	pleasure
chcieć skończyć	to want to end [something]	przyjmować do wiadomości	to acknowledge
chcieć wydać dźwięk	to want to make a sound	przykrość	unpleasantness, hurt
chcieć zainteresować	to want to interest [someone]	przymulać się	to feel brain-fogged
chcieć zrobić	to want to do [something]	radość	happiness
chcieć żeby ktoś skończył mówić	to want for someone to stop talking	relaksować się	to relax
chytrość	cunningness	rozbawienie	amusement
coś NEG podoba się	something is not to [his/her] liking	rozdwojenie	ambivalence
coś NEG pasuje	something does not fit	rozgoryczenie	bitterness
denerwować się	to get nervous/to get angry	rozpacz	despair
dezaprobata	disapproval	satysfakcja	satisfaction
dezorientacja	disorientation	skrucha	remorse
docenienie	appreciation	skupienie	focus
domyślać się	to guess	smutek	sadness
duma	pride	spieszyć się	to be in a hurry
zdziwienie	surprise, bewilderment	spokój	calm
ekscytacja	excitement	strach	fear
entuzjazm	enthusiasm	szczęście	joy
euforia	euphoria	szok	shock
fascynacja	fascination	unikanie	avoidance
gniew	anger	uspokajać się	to calm oneself down
irytacja	irritation	uważać	to be careful
konsternacja	consternation	uznanie	appreciation
krzywda	hurt	wahać się	to hesitate
ledwo kontaktować	to be barely responsive	wątpienie	doubt
lekceważyć	to disregard	wina	fault
lęk	anxiety	wkurzyć się	to get mad
mieć dość	to be fed up	wpaść na pomysł	to come up with an idea
mieć inne zdanie	to have a different opinion	wrogość	hostility
myśleć	to think	wstręt	disgust
naburmuszona	grumpy	wstyd	shame
napięcie	tension	wściekłość	rage
NEG akceptować	to not accept	wymyślić coś	to think of something
negatywna emocja	negative emotion	wyższość	superiority
NEG chcieć	to not want	zachwyt	admiration
NEG chcieć trzymać	to not want to hold	zaciekawienie	interest
NEG-dobre	not good	zaciętość	stubbornness
NEG emocji	no emotions	zadowolenie	contentment
NEG mieć na coś słów	to lack words	zainteresowanie	interest
NEG-pewność	uncertainty	zakłopotanie	embarrassment
NEG rozumieć	to not understand	zalotna	flirtatious
NEG spodziewać się	to not expect	załamanie	devastation
NEG-szczera	insincere	zaskoczenie	surprise
NEG-śmiałość	shyness	zasłuchanie	a state of listening very closely
NEG wiedzieć	to not know	zastanawiać się	to wonder
NEG wierzyć	to not believe	zauroczony	charmed
NEG-zadowolenie	dissatisfaction	zawieszona	baffled, paralyzed
NEG-zdecydowanie	undecidedness	zażenowanie	embarrassment
NEG zgadzać się	to not agree	zdołowana	feeling down
neutralny	neutral	zgadzać się	to agree
NEG-chęć	reluctance	zła	angry/bad/evil
NEG-pokój	anxiety	złe emocje	bad emotions
NEG-smak	distaste	złość	anger
NEG-swojo	uncomfortably	zmęczenie	tiredness
NEG-zainteresowana	uninterested	zniecierpliwienie	impatience
obojętność	indifference	znudzenie	boredom
obrzydzenie	disgust	zobaczyć	to see
odraza	disgust	zostawiać coś bez komentarza	to leave something without comment
ohyda	vileness, repulsion	żal	regret, sorrow

Supplementary Table S12

Translations of Maniq responses used in the cluster analysis

A list of Maniq responses used in the cluster analysis, their translations, and classification

Maniq response	English translation	Response type (MS - 'mental state'; BA- 'bodily action'; O - 'other')
munaʔ	to be happy	MS
pikluk	to smile, to laugh	BA
baʔɛt	to be good, happy	MS
NEG kahey	to not cry	BA
NEG yɔʔ	to not be angry	MS
yikieh	to part lips, usually also showing teeth	BA
NR	(no response)	O
nɨk	to think, to feel	MS
dɛŋ	to see, to look	BA
NEG gəbaʔ	to not be angry	MS
lep	to close or move upper eyelid	BA
ʔɔt	to miss, to lack	MS
haʔɨp	to know, to remember	MS
NEG ʔiyay	to not be sad, irritated, upset	MS
ʔiyay	to be sad, irritated, upset	MS
NEG dɛŋ	to not see, to not look	BA
lŋyeŋ	to make white (e.g., of wide open eyes)	BA
hkhik	to get startled, to have a startle reaction	BA
biʔuɲ	to open one's eyes widely	BA
hah	to open one's mouth	BA
biyɔh	to talk	BA
blahut	to be veiled with white clouding or tears (of eyes)	BA
kayeŋ	to hear, to listen	BA
pəntɛw	to look ahead, to direct one's gaze forward	BA
kahey kalɲɛs	to be crying inside ('in one's heart')	MS
lɨk	to swallow	BA
gəbaʔ	to be angry	MS
yɔʔ	to be angry	MS
citũn	to raise upper lip	BA
kiɲus	to wrinkle nose	BA
haʔɔ̃ŋ	to be brown/gray	BA
hiɲɛm	to squint	BA
kipuʔ	to close, to press together	BA
bakãt	to cry	BA
yihɛŋ	to widen one's nostrils	BA
ɲup	to close or tighten lower eyelid	BA
lkcɔk	to cause pain (usually of backache or stomachache)	BA
kahey	to cry	BA
ciʔut	to wrinkle	BA
ʔantɨŋ	to be afraid	MS
klatis	to turn mouth corners down	BA
NEG haʔɨp	to not know, to not remember	MS
cabɨc	to be teary	BA
ŋɔk nay	to sit/stay alone	BA
kitũt	to bulge, to protrude	BA
cawɛ	to be lopsided, assymetrical	BA

Supplementary Table S13

Translations of Polish responses used in the cluster analysis

A list of Polish responses used in the cluster analysis, their translations, and classification

Polish response	English translation	Response type (MS - 'mental state'; BA- 'bodily action'; O - 'other')
znudzenie	boredom	MS
zastanawiać się	to wonder	MS
myśleć	to think	MS
obojętność	indifference	MS
patrzeć	to look	BA
zdziwienie	surprise, bewilderment	MS
zniesmaczenie	distaste	MS
coś się NEG podoba	something is not to [his/her] liking	MS
NEG mina	to not [make] a face	BA
złość	anger	MS
smutek	sadness	MS
przykrość	unpleasantness, hurt	MS
wstyd	shame	MS
NEG-śmiałość	shyness	MS
zakłopotanie	embarrassment	MS
radość	happiness	MS
zadowolenie	contentment	MS
szczęście	joy	MS
zaskoczenie	surprise	MS
strach	fear	MS
przerażenie	terror	MS
uśmiech	smile	BA
płacz	cry	BA
wątpienie	doubt	MS
irytacja	irritation	MS
niepokój	anxiety	MS
wstręt	disgust	MS
obrzydzenie	disgust	MS
NR	(no response)	O
zażenowanie	embarrassment	MS
dezaprobata	disapproval	MS
żal	regret, sorrow	MS
zachwyt	admiration	MS
rozbawienie	amusement	MS
NEG-zadowolenie	dissatisfaction	MS
nic	nothing	O
odraza	disgust	MS
satysfakcja	satisfaction	MS
neutralnie	neutrally	MS
szok	shock	MS
NEG spodziewać się	to not expect	MS
NEG-pewność	uncertainty	MS
oczekiwać	to await	MS
spokój	calm	MS
zasłuchanie	a state of listening very closely	MS
słuchać	to listen	BA
pewność siebie	self-confidence	MS
rozpacz	despair	MS
NEG wierzyć	to not believe	MS
NEG wiedzieć	to not know	MS
duma	pride	MS
NEG zgadzać się	to not agree	MS
gniew	anger	MS
zaciekawienie	interest	MS
przygęnbienie	dejection	MS
podziw	admiration	MS

References

1. Wnuk, E. *Semantic Specificity of Perception Verbs in Maniq.* (Radboud University, 2016).
2. Townsend, C. Egalitarianism, evolution of. in *The International Encyclopedia of Anthropology* 1–7 (American Cancer Society, 2018).
3. Statistics Poland. *National Population and Housing Census 2021 Population. Size and Demographic-Social Structure in the Light of the 2021 Census Results.* (2023).
4. Urząd Statystyczny w Gdańsku. *Kształcenie Dorosłych w 2016 r.* (2018).
5. Majid, A., Bowerman, M., Staden, M. van & Boster, J. S. The semantic categories of cutting and breaking events: A crosslinguistic perspective. *Cogn. Linguist.* **18**, 133–152 (2007).
6. Majid, A. & Burenhult, N. Odors are expressible in language, as long as you speak the right language. *Cognition* **130**, 266–270 (2014).
7. Majid, A. *et al.* Differential coding of perception in the world's languages. *Proc. Natl. Acad. Sci.* **115**, 11369–11376 (2018).
8. Wnuk, E., Verkerk, A., Levinson, S. C. & Majid, A. Color technology is not necessary for rich and efficient color language. *Cognition* **229**, 105223 (2022).
9. Huisman, J. L. A., Hout, R. van & Majid, A. Cross-linguistic constraints and lineage-specific developments in the semantics of cutting and breaking in Japonic and Germanic. *Linguist. Typology* **27**, 41–75 (2023).
10. Mead, M. *Coming of Age in Samoa: A Psychological Study of Primitive Youth for Western Civilisation*. (W. Morrow & Company, 1928).
11. Porath, N. Foraging Thai literary culture: A performing tribe of South Thailand. in *Senri Ethnological Studies* 117–138 (National Museum of Ethnology, 2001).
12. Howell, S. Rules not words. in *Indigenous psychologies: The anthropology of the self* (eds. Heelas, P. & Lock, A.) 133–143 (Academic Press, 1981).
13. Stasch, R. Knowing minds is a matter of authority: Political dimensions of opacity statements in Korowai moral psychology. *Anthropol. Q.* **81**, 443–453 (2008).
14. Kirmayer, L. J. Psychotherapy and the cultural concept of the person. *Transcult. Psychiatry* **44**, 232–257 (2007).
15. Geertz, C. Person, time, and conduct in Bali. in *The Interpretation of Cultures: Selected Essays* 360–411 (Basic Books, 1973).
16. Shweder, R. A. & Bourne, E. J. Does the concept of the person vary cross-culturally? in *Cultural Conceptions of Mental Health and Therapy* (eds. Marsella, A. J. & White, G. M.) 97–137 (Springer Netherlands, 1982).
17. Kricheff, D. A. *Action and Identity Among Maniq People in Southern Thailand*. (University College London, 2019).
18. Astuti, R. "The Vezo are not a kind of people": identity, difference, and "ethnicity" among a fishing people of western Madagascar. *Am. Ethnol.* **22**, 464–482 (1995).

19. Russel, J. Is there universal recognition of emotion from facial expression? A review of the cross-cultural studies. *Psychol. Bull.* **115**, 102 (1994)
20. Gendron, M. *et al.* Emotion perception in Hadza hunter-gatherers. *Sci. Rep.* **10**, 3867 (2020)
